# Impact of professional nursing interventions on clinical outcomes in patients with acute gastric bleeding: a retrospective analysis

**DOI:** 10.1038/s41598-024-55558-9

**Published:** 2024-03-01

**Authors:** Xueqin Yuan, Fang Yu, Shouzhi Fu

**Affiliations:** Department of ICU/Emergency, Wuhan Third Hospital, Tongren Hospital of WuHan University, Wuhan, 430074 Hubei China

**Keywords:** Gastric bleeding, Emergency nursing, Clinical outcomes, Nursing intervention, Disease prevention, Health services, Public health

## Abstract

Acute gastric bleeding (AGB) is a common and potentially serious complication in patients with gastrointestinal disorders. Nursing interventions play a critical role in the management of acute gastric bleeding, but their impact on clinical outcomes is not well understood. The aim of this retrospective analysis was to evaluate the impact of nursing interventions on clinical outcomes in patients with acute gastric bleeding. A retrospective review of medical records was conducted for 220 patients with acute gastric bleeding who were admitted to the hospital between February 2022 and February 2023. Patients were divided into two groups based on whether or not they received nursing interventions during their hospital stay. Clinical outcomes, including length of hospital stay, blood transfusion requirements, and mortality rates, were compared between the two groups using descriptive statistics and logistic regression analysis. Of the 220 patients included in the study, 168 (76.4%) received nursing interventions during their hospital stay. Patients who received nursing interventions had a significantly shorter length of hospital stay (mean = 7.2 days, SD = 2.1) compared to those who did not receive nursing interventions (mean = 10.5 days, SD = 3.4, *p* < 0.001). Additionally, the 90-day mortality rate was lower in the group receiving professional nursing interventions (4.2% vs. 15.4%, *p* = 0.010). Fewer patients who received nursing interventions required blood transfusions (33.3% vs. 65.2%, *p* < 0.001) and mortality rates were lower (6.7% vs. 20.8%, *p* = 0.04). Multivariate logistic regression analysis suggested that professional nursing intervention was a protective factor for postoperative rebleeding in patients with gastric hemorrhage (OR 0.727, 95% CI 0.497–0.901, *P* < 0.001). The results of this retrospective analysis suggest that nursing interventions are associated with improved clinical outcomes in patients with acute gastric bleeding. The implementation of nursing interventions, such as individualized care plans, monitoring and evaluation, and patient education, should be encouraged to optimize patient outcomes in this population. Further research is needed to identify the most effective nursing interventions and to evaluate their cost-effectiveness.

## Introduction

Acute gastric bleeding (AGB) is a common and serious complication in patients with gastrointestinal disorders. It typically requires urgent medical care and may lead to a significant incidence and mortality rate, especially in patients with ulcers, inflammation, or other gastric issues. Early symptoms may include melena, hematemesis, abdominal pain, etc. The detection of AGB usually involves procedures such as endoscopy and imaging studies to identify the source of bleeding and assess the extent of pathology. Treatment modalities encompass drug therapy, endoscopic hemostasis, etc., depending on the cause of bleeding and individual patient conditions^1,2^. In recent years, nursing interventions have been increasingly recognized as an important part of the management of AGB^3^. Nurses play a crucial role in the management of patients with Acute Gastric Bleeding (AGB), including closely monitoring vital signs, early identification, and timely reporting of bleeding, as well as assisting physicians in treatment. However, there is still limited evidence on the impact of nursing interventions on clinical outcomes in this patient population.

Some studies have suggested that nursing interventions, such as close monitoring of vital signs, early identification of bleeding, and timely administration of medications, may improve patient outcomes in AGB. For example, A study by Li et al.^4^ found that tripartite intensive care significantly reduced the rate of bleeding during hospitalization, as well as reducing the rate of complications, promoting recovery, and improving the quality of life of the patients. Providing care interventions for all patients is crucial, but specific diseases require corresponding specialized nursing interventions to achieve optimal outcomes.

Despite these promising findings, there is still a lack of consensus on the most effective nursing interventions and their impact on clinical outcomes in patients with AGB^5,6^. Therefore, further research is needed to evaluate the effectiveness of different nursing interventions and to identify factors that may influence their impact on patient outcomes^7–9^. The present retrospective analysis aimed to evaluate the impact of nursing interventions on clinical outcomes in patients with AGB. We hypothesized that nursing interventions would be associated with improved clinical outcomes, such as shorter hospital stays, fewer blood transfusions, and lower mortality rates. To test this hypothesis, we conducted a retrospective review of medical records for 220 patients with AGB who were admitted to the hospital between February 2022 and February 2023. Patients were divided into two groups based on whether or not they received nursing interventions during their hospital stay. Clinical outcomes were compared between the two groups using descriptive statistics and logistic regression analysis.

By identifying the impact of nursing interventions on clinical outcomes in patients with AGB, this study could provide important insights to guide the development of effective nursing interventions in this patient population. Our primary outcomes include the duration of hospitalization, the proportion of patients requiring transfusion, the occurrence rate of rebleeding, and postoperative complications such as infections, coagulation issues, etc. Secondary outcomes comprise the readmission rates at 30 and 90 days. Furthermore, it could help to enhance our understanding of the complex interplay between nursing interventions and clinical outcomes in patients with AGB. The hypothesis of the study is that professional nursing intervention can improve the primary and secondary outcomes of patients with AGB.

## Materials and methods

### Patient selection study design and participants

This study employed a retrospective cohort study design to evaluate the effectiveness of nurse-led interventions in improving outcomes in patients with acute gastric bleeding (AGB). The study participants consist of adult patients (18 years and above) who presented to the emergency department and were diagnosed with AGB, requiring hospitalization for intervention, during a specific time period (e.g., February 2022 to February 2023). The decision for patients to undergo professional nursing interventions is a joint decision between family members and the healthcare team. Both family members and patients have a high degree of autonomy in the decision-making process. AGB is defined as sudden bleeding in the stomach, with causes including gastric ulcers, gastric cancer, and other related conditions. The diagnosis of AGB was determined by collaboration between gastrointestinal surgeons, emergency medicine specialists, and radiologists based on clinical symptoms, X-ray examinations, and gastroscopy reports.

Strict inclusion and exclusion criteria were applied to ensure the integrity of the cases. Inclusion criteria were as follows: (1) age 18 years or above at the time of diagnosis, (2) confirmed diagnosis of AGB through imaging studies and physical examination, (3) absence of significant comorbidities (Including severe cardiovascular diseases, etc.), and (4) first-time occurrence of AGB. Exclusion criteria were: (1) incomplete clinical data, (2) death within 24 h of hospital admission, and (3) history of previous surgeries. This research was performed in accordance with the Declaration of Helsinki and was approved by the Ethics Committee of Affiliated Kunshan Hospital of Jiangsu University [Approve number TH-20211127].

### Assessment of severity in acute gastric bleeding

The severity of gastric bleeding is determined based on factors such as the volume of bleeding, rate of bleeding, clinical symptoms, and the presence of complications.

#### Volume of bleeding

Gastric bleeding refers to damage to the gastric mucosa, leading to localized vascular rupture and bleeding. If the patient experiences a relatively small volume of bleeding, the condition is generally not considered particularly severe. However, if the bleeding volume is substantial, it may lead to shock, indicating a more serious situation.

#### Rate of bleeding

If the bleeding occurs at a slow rate and the volume is relatively small, the condition is generally not considered particularly severe. Conversely, a faster rate of bleeding, coupled with a larger volume, is indicative of a more severe condition.

#### Clinical symptoms

The absence of significant discomfort symptoms in the patient generally suggests a less severe condition. On the other hand, the presence of symptoms such as vomiting, melena, fever, etc., usually indicates a more severe situation.

#### Presence of complications

A smaller volume of bleeding without evident complications is typically not considered particularly severe. However, if the bleeding volume is larger and complications are present, the condition is likely to be more serious.

### Sample size and power calculation

Firstly, we utilized experiential judgment for multivariate analysis based on previous BMJ literature^10^. According to the literature, we calculated the number of endpoint events by taking five times the number of independent variables. With 19 independent variables, this equates to 95. In other words, there should be 95 patients with endpoint events. The number of patients recruited in our study who subsequently reached endpoint events exceeds 95, thus confirming this experiential judgment.

Secondly, we employed PASS (version: 11.0) for calculation. Within PASS, we utilized the "Regression" section under "Logistics Regression," setting a two-sided Alpha of 0.05 and Beta of 0.1. The corresponding results were incorporated into the PASS software, yielding a one-sided Sample Size (N) of 33. The sample size we included is greater than 66.

### Professional nursing interventions

The non-professional nursing intervention group, serving as the control group, received standard nursing care. This care included the provision of explanations regarding the current treatment methods and their purposes to facilitate patient understanding of the treatment measures. We assisted patients in identifying potential problems that may arise during the treatment process and provided guidance on precautions to be taken. Standard nursing care also involved the continuous monitoring of relevant indicators throughout the treatment period, with prompt communication to the attending physician in case of any abnormalities. The control group received patient education on proper nutrition and medication use, covering information about potential adverse drug reactions and preventive measures.

It is important to note that electrolyte monitoring was a part of the standard nursing care provided to the control group, ensuring the comprehensive evaluation of patients' health status during the treatment period. This approach aims to capture a realistic representation of routine nursing practices in a standard care setting.

The group receiving professional nursing interventions is provided with high-quality professional nursing services delivered by general nurses., including the following aspects:

Psychological care: Many patients with gastric bleeding have misunderstandings about their disease and may experience negative emotions that can aggravate their condition. Therefore, nursing staff should closely monitor patients' psychological changes, provide education on gastric bleeding and its treatment, and offer emotional support to help patients stabilize their emotions and voluntarily cooperate with healthcare personnel. Family members can also be given appropriate nursing guidance to provide effective support for the patient. Fluid intervention: After admission, intravenous access is quickly established, electrolyte changes are tested, and vital signs are closely monitored, including body temperature, pulse, respiration, blood pressure, and other indicators. The amount of vomiting blood, black stool, and 24-h in and out volume are recorded, and hemostatic drugs are administered if necessary. In cases of shock, sodium bicarbonate or saline is used to expand volume, and blood transfusion is administered as needed. For patients with cirrhotic portal hypertension, caution must be taken to avoid rehemorrhage due to increased portal vein pressure from further blood transfusion. The amount of fluid input should be reduced appropriately to avoid excessive input, resulting in acute edema and rehemorrhage. Life care: Patients with acute gastric hemorrhage require strict bed rest, with no food or drink allowed. Electrolyte imbalances must be corrected, and attention should be paid to maintaining an appropriate temperature. Oxygen therapy should be administered to patients with severe bleeding. Diet care: Patients with gastric bleeding should be given a light diet, with regular and moderate intake, and small meals. Patients with nausea and vomiting should be instructed to fast until symptoms disappear and bleeding stops before resuming a proper diet. Exercise care: After the patient's condition stabilizes, exercise should be encouraged to improve body resistance. However, the amount of exercise should follow the principle of gradual progress to avoid sudden increases. Health education: Patients with gastric bleeding should receive detailed information about their disease and preventive measures. For patients with recurring gastric bleeding, education should focus on avoiding causative factors and preventing complications to ensure better control of the condition and self-care.

### Nurses' propaganda and education details in professional nursing interventions

Assess patient's understanding and emotional state: Before beginning the mission, nursing staff should talk with patients to find out how much they know about gastric bleeding as well as any misconceptions and anxiety they may have.

Provide education on gastric bleeding and its treatment: Nursing staff should provide patients and their families with basic knowledge about gastric bleeding, including information about causes, symptoms, diagnostic methods, treatment options, and prognosis.

Emphasize the importance of treatment and cooperation: Nursing staff should clearly inform patients of the importance of their treatment and encourage them to actively cooperate with their healthcare provider's treatment plan.

Offer emotional support and reassurance: Since gastric hemorrhage may lead to negative emotions, nursing staff should listen patiently to patients' emotional expressions and understand and respect their feelings.

Instruct family members on effective support methods: Nursing staff can provide guidance to families on how to effectively support the patient, including emotional support, dietary care, and daily living care.

Highlight dietary and lifestyle recommendations: Nursing staff should introduce patients to appropriate diets and lifestyles to help them better manage and prevent stomach bleeding. This includes advice on dietary precautions, meal plans, and regular routines.

Guide rehabilitation exercises gradually: After the patient's condition is stabilized, nursing staff can recommend appropriate rehabilitation exercises to help improve their body's resistance and ability to recover.

Educate on prevention and self-management: For patients with recurrent gastric bleeding, nursing staff should focus on teaching how to avoid triggers and prevent complications to ensure better control and self-care.

Ensure patient's comprehension and application of education: Nursing staff should double-check that patients understand the information provided during the education process and encourage them to apply the knowledge and skills they have learned in real-life situations.

Follow up regularly and address new concerns: Nursing staff should regularly follow up on patients' conditions and treatment progress, answer any new questions they may have in a timely manner, and make any necessary adjustments and additional teachings. In summary, it is important to note that professional nursing interventions are designed to assist and support the treatment plans of the medical team. Nursing interventions are not intended to replace medical treatments but rather to work collaboratively with the physician's treatment, aiming to comprehensively enhance the quality of patient care and treatment outcomes. And both professional nursing interventions and non-professional nursing interventions are provided by general nurses.

### Data collection and outcome measures

Data collection for this retrospective analysis involved reviewing medical records of 220 patients with acute gastric bleeding who were admitted to the hospital between February 2022 and February 2023. The following outcome measures were assessed to evaluate the impact of nursing interventions on clinical outcomes:

Length of Hospital Stay: The length of hospital stay was recorded for each patient and compared between the group that received nursing interventions and the group that did not.

Blood Transfusion Requirements: The number of patients requiring blood transfusions was documented for both groups. A comparison was made to determine if nursing interventions had an influence on blood transfusion requirements.

Occurrence of Rebleeding: The incidence of rebleeding, defined as a rebleeding episode that occurs after initial treatment and complete hemostasis, was recorded and compared between the groups that received the nursing intervention and the group that did not receive the nursing intervention.

Mortality Rates: Mortality rates were calculated for patients in both groups to assess the impact of nursing interventions on patient survival.

### Data analysis

The data collected from medical records served as the foundation for analyzing and comparing outcome measures between the group that received nursing interventions and the group that did not. Descriptive statistics, including means and standard deviations, were calculated to summarize the data and identify any significant differences. Covariates entered into the multivariate logistic regression analysis were prespecified, and the association between nursing interventions and postoperative rebleeding was assessed while considering potential confounding factors.

All statistical analyses were performed using SPSS 25.0 (IBM, Armonk, New York, USA). A significance level of *P* < 0.05 (two-sided) was considered statistically significant. To visually present the results, graphs were created using R language (version 4.0.5) and GraphPad Prism (version: 8.0). Sample size estimation was conducted before the study using PASS (version: 11.0).

Multivariate analysis included the consideration of prespecified covariates to account for potential confounding effects. Specifically, factors such as age, severity of bleeding, and history of gastrointestinal disorders were entered into the logistic regression model to assess their impact on the association between nursing interventions and postoperative rebleeding.

### Follow-up

Following discharge from the hospital, all patients were followed up by two trained professionals. They conducted phone interviews with the patients to inquire about their current health status and requested them to visit the hospital for a comprehensive review. During the follow-up period, the professionals collected information regarding any ongoing symptoms, recurrence of gastric bleeding, or other complications. They also assessed the need for additional interventions or treatments. The collected data from the follow-up interviews were analyzed to determine the long-term impact of nursing interventions on patient outcomes, including the recurrence of bleeding and the overall effectiveness of the care provided. The follow-up continued until six months after the patients were discharged.

### Ethical approval and consent to participation

Informed consent was obtained from all subjects and/or their legal guardian(s). This research was performed in accordance with the Declaration of Helsinki and was approved by the Ethics Committee of Affiliated Kunshan Hospital of Jiangsu University [Approve number TH-20211127].

## Results

### Baseline information on acute gastric bleeding with and without skilled nursing interventions

**Figure 1:**
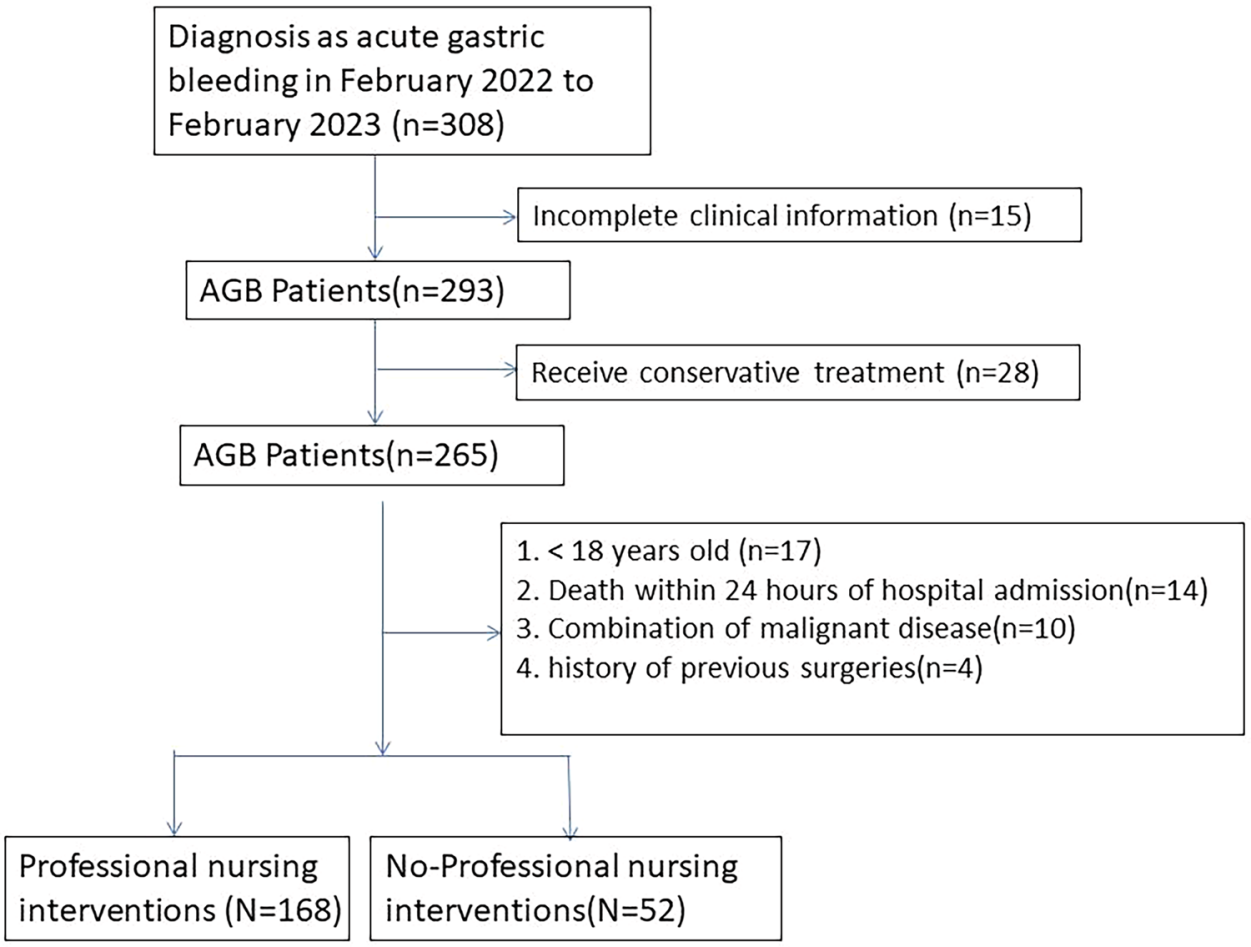
Inclusion and exclusion table for AGB patients.

The inclusion and exclusion criteria for this study are outlined in Fig. 1. There were 168 patients who received Professional nursing intervention and 52 patients who did not receive skilled nursing intervention. All patients were sourced from the general ward. No significant differences were found between the groups in terms of gender, age, BMI, history of gastrointestinal disorders, hypertension, diabetes, coronary artery disease, severity of bleeding, platelet count, and endoscopy findings. Patients who received professional nursing interventions had higher hemoglobin levels (9.8 ± 1.7 g/dL vs. 8.9 ± 2.0 g/dL, *p* < 0.001) and lower INR values (1.5 ± 0.3 vs. 1.6 ± 0.4, *p* = 0.030). Regarding clinical outcomes, patients with professional nursing interventions had shorter hospital stays (7.2 ± 2.1 days vs. 10.5 ± 3.4 days, *p* < 0.001), lower blood transfusion requirements (32.7% vs. 65.4%, *p* < 0.001), and lower rebleeding rates (3.6% vs. 9.6%, *p* < 0.001). In summary, professional nursing interventions were associated with higher hemoglobin levels, lower INR values, shorter hospital stays (mean difference: 3.3 days), reduced blood transfusion requirements (risk ratio: 0.50), and lower rebleeding rates (risk ratio: 0.37) in patients with acute gastric bleeding (Table 1).

**Table 1:** Baseline information of patients with acute gastric bleeding at admission (n = 220).

	Professional nursing interventions (n = 168)	No-professional nursing interventions (n = 52)	*P*-value^‡^
Gender			0.790
Male	104 (61.9)	31 (59.6)	
Female	64 (38.1)	21 (40.4)	
Age			0.634
< 60 y	92 (54.8)	26 (50.0)	
≥ 60 y	76 (45.2)	26 (50.0)	
BMI			0.634
< 25 kg/m^2^	97 (57.7)	28 (53.8)	
≥ 25 kg/m^2^	71 (42.3)	24 (46.2)	
History of gastrointestinal disorders			0.490
No	93 (55.3)	52 (50.0)	
Yes	75 (44.7)	52 (50.0)	
Hypertension			0.550
No	102 (60.7)	34 (65.4)	
Yes	66 (39.3)	18 (34.6)	
Diabetes			0.811
No	126 (75.0)	38 (73.1)	
Yes	42 (25.0)	14 (26.9)	
Coronary artery disease			0.672
No	141 (83.9)	45 (86.5)	
Yes	27 (16.1)	7 (13.5)	
Severity of bleeding			0.481
Mild	47 (28.0)	12 (23.1)	
Moderate	80 (47.6)	26 (50.0)	
Severe	41 (24.4)	14 (26.9)	
Hemoglobin level			
g/dL	9.8 ± 1.7	8.9 ± 2.0	< 0.001
Platelet count			
× 10^3/μL	188 ± 54	174 ± 41	0.123
INR	1.5 ± 0.3	1.6 ± 0.4	0.030
Endoscopy findings			0.354
Ulcer	74 (44.0)	28 (53.8)	
Erosion	54 (32.1)	13 (25.0)	
Varices	31 (18.5)	8 (15.4)	
Treatment modalities			0.576
Endoscopic therapy	141 (83.9)	39 (75.0)	
Surgical intervention	27 (16.1)	10 (19.2)	
Conservative treatment	0 (0.0)	3 (5.8)	
Length of hospital stay			
Days	7.2 ± 2.1	10.5 ± 3.4	< 0.001
Blood transfusion requirements			< 0.001
No	113 (67.3)	18 (34.6)	
Yes	55 (32.7)	34 (65.4)	
Rebleeding			< 0.001
No	162 (96.4)	47 (90.4)	
Yes	6 (3.6)	5 (9.6)	

The values in parentheses are percentages unless indicated otherwise.

*AGB* Acute gastric bleeding.

^‡^χ^2^ test with Yates’ correction.

^§^Sharps injuries, firearm injuries, etc.

^‡^Use of anticoagulants or antiplatelet drugs.

### Comparison of AGB complications in professional nursing interventions group and no-professional nursing interventions

There were some differences in perioperative complications between the group with Professional nursing intervention (n = 168) and the group without Professional nursing intervention (n = 52). Significant differences were observed in perioperative complications, including rebleeding of the stomach (*p* < 0.001), coagulation complications (*p* < 0.001), and infection (*p* = 0.003). Patients who received professional nursing interventions had lower rates of rebleeding (10.7% vs. 32.7%) and lower rates of coagulation complications (1.2% vs. 15.4%) compared to those without professional nursing interventions. Rates of stomach pain and bloating were similar between the groups (*p* = 0.160), while no significant differences were found in non-gastrointestinal complications, including circulatory (*p* = 1.000), respiratory (*p* = 0.596), and urinary complications (*p* = 1.000). Furthermore, patients who received professional nursing interventions had significantly lower rates of infection (0.6% vs. 9.6%) compared to those without professional nursing interventions (Table 2). The primary source of patient infections is hospital-acquired.

**Table 2:** Comparison of AGB complications in Professional Nursing Interventions group and No- Professional Nursing Interventions (n = 220).

	Professional nursing interventions (n = 168)	No-professional nursing interventions (n = 52)	*P*-value^‡^
Perioperative complication			< 0.001
Rebleeding of the stomach			
No	150 (89.3)	35 (67.3)	
Yes	18 (10.7)	17 (32.7)	
Stomach pain and bloating			0.160
No	161 (95.8)	47 (90.4)	
Yes	7 (4.2)	5 (9.6)	
Non-gastrointestinal complication			
Circulatory			1.000
No	166 (98.8)	52 (98.1)	
Yes	2 (1.2)	1 (1.9)	
Respiratory			0.596
No	165 (98.2)	51 (96.2)	
Yes	3 (1.8)	2 (3.8)	
Urinary			1.000
No	168 (100.0)	52 (100.0)	
Yes	0 (0.0)	0 (0.0)	
Coagulation			< 0.001
No	166 (98.8)	44 (84.6)	
Yes	2 (1.2)	8 (15.4)	
Infection			0.003
No	167 (99.4)	47 (90.4)	
Yes	1 (0.6)	5 (9.6)	

The values in parentheses are percentages unless indicated otherwise.

*AGB* Acute gastric bleeding.

^‡^χ^2^ test or Fisher's test.

### Univariate and multivariate cox proportional hazards regression analyses of rebleeding in AGB patients undergoing radical therapy

In the univariate analysis, Age (*p* = 0.031), history of gastrointestinal disorders (*p* = 0.008), severity of bleeding (*p* < 0.001), and Professional Nursing Interventions (*p* < 0.001) were significantly associated with rebleeding. In the multivariate analysis, Age (*p* < 0.001), history of gastrointestinal disorders (*p* = 0.012), severity of bleeding (*p* < 0.001), and Professional Nursing Interventions (*p* < 0.001) remained significant predictors of rebleeding.

Specifically, patients aged < 60 years had a lower risk of rebleeding compared to those aged ≥ 60 years (OR 1.187, 95% CI 1.076–1.302). Patients without history of gastrointestinal disorders had a lower risk of rebleeding compared to those with history of gastrointestinal disorders (OR 1.288, 95% CI 1.091–1.479). Patients with mild severity of bleeding on admission had a lower risk of rebleeding compared to those with moderate or severe severity of bleeding (moderate: OR 1.213, 95% CI 1.155–1.431; severe: OR 1.455, 95% CI 1.210–1.708). Patients who received professional nursing interventions had a lower risk of rebleeding compared to those who did not (OR 0.727, 95% CI 0.497–0.901).

Other variables such as gender, BMI, Hypertension, Diabetes, Coronary Artery Disease, Endoscopy Findings, and Treatment Modalities were not significantly associated with rebleeding (Table 3).

**Table 3:** Univariate and multivariate logistic regression analysis of risk factors associated with Rebleeding in all AGB patients.

Variables	Univariate analysis			Multivariate analysis		
	*P*	OR	95% CI	*P*	OR	95% CI
Gender	0.315					
Male		Ref	–			
Female		0.813	0.587–1.342			
Age (years)	0.031			< 0.001		
< 60 y		Ref	–		Ref	–
≥ 60 y		1.230	1.060–1.721		1.187	1.076–1.302
BMI	0.588					
< 25 kg/m^2^		1.213	0.891–1.381			
≥ 25 kg/m^2^		Ref	–			
History of gastrointestinal disorders	0.008			0.012		
No		Ref	–		Ref	–
Yes		1.372	1.109–1.531		1.288	1.091–1.479
Hypertension	0.611					
No		Ref	–			
Yes		1.051	0.916–1.264			
Diabetes	0.132					
No		Ref	–			
Yes		1.146	0.899–1.364			
Coronary artery disease	0.329					
No		Ref	–			
Yes		1.178	0.842–1.415			
Severity of bleeding	< 0.001			< 0.001		
Mild		Ref	–		Ref	–
Moderate		1.134	1.087–1.366		1.213	1.155–1.431
Severe		1.376	1.198–1.516		1.455	1.210–1.708
Endoscopy findings	0.692					
Ulcer		Ref	–			
Erosion		1.089	0.781–1.669			
Varices		0.816	0.431–1.390			
Treatment modalities	0.288					
Endoscopic therapy		Ref	–			
Surgical intervention		0.924	0.746–1.365			
Professional nursing interventions	< 0.001			< 0.001		
No		Ref	–		Ref	–
Yes		0.613	0.378–0.869		0.727	0.497–0.901

*OR* Odds ratios; *AGB* Acute gastric bleeding.

### Comparison of the prognosis of patients with acute gastric bleeding in the professional nursing intervention group and the group without professional nursing intervention

The readmission rate for patients who received skilled nursing interventions was 8.3%, which was significantly lower than the 21.2% for patients who did not receive skilled nursing interventions (*P* = 0.021); the 30-day mortality rate for patients who also received skilled nursing interventions was 3.6%, whereas the 30-day mortality rate for those who did not receive skilled nursing interventions was slightly higher at 5.8%, this difference was not statistically significant (*P* = 0.444). However, patients who received skilled nursing interventions had a significantly lower 90-day mortality rate of 4.2%, while patients who did not receive skilled nursing interventions had a higher 90-day mortality rate of 15.4%. The difference in 90-day mortality rate was statistically significant (*P* = 0.010) (Table 4).

**Table 4:** Comparison of clinical prognostic of AGB between Professional nursing intervention group and no Professional nursing intervention group (n = 220).

	Professional nursing interventions (n = 168)	No-professional nursing interventions (n = 52)	*P*-value^‡^
Readmission rate			
Readmission numbers (%)	14 (8.3)	11 (21.2)	0.021
30-day mortality			
Death numbers (%)	6 (3.6)	3 (5.8)	0.444
90-day mortality			
Death numbers (%)	7 (4.2)	8 (15.4)	0.010

*AGB* Acute Gastric Bleeding.

### Effectiveness of integrated nursing interventions on 30-day mortality, 90-day mortality and readmission rates

**Figure 2:**
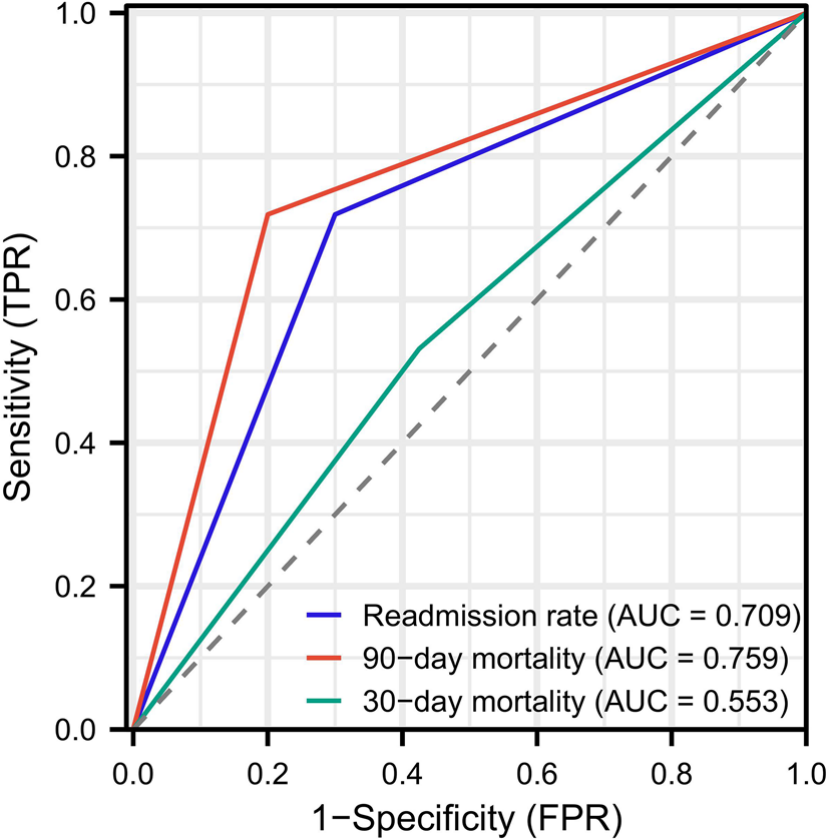
Effectiveness of professional nursing interventions on 30-day mortality, 90-day mortality and readmission rates.

ROC curves were employed to assess the effectiveness of skilled nursing interventions in predicting short-term clinical prognosis in patients with traumatic brain injury. The ROC curves depicted the relationship between sensitivity and specificity for the prediction of 30-day mortality, 90-day mortality, and readmission rates resulting from skilled nursing interventions. The horizontal axis represented specificity, while the vertical axis indicated sensitivity. The results revealed that the area under the curve (AUC) was 0.553 and 0.709 for the prediction of 30-day mortality and readmission rates, respectively (see Fig. 2). For the 90-day mortality rate, the AUC was found to be 0.759.

## Discussion

Acute gastric bleeding (AGB) is a significant complication in patients with gastrointestinal disorders, necessitating urgent medical attention and potentially leading to high morbidity and mortality rates^11,12^. Professional nursing interventions by general nurses also play a crucial role in aiding the prognosis of AGB patients, and an increasing number of individuals recognize that nursing interventions are equally critical in the treatment of AGB. This retrospective analysis aims to evaluate the impact of Professional nursing interventions by general nurses on the clinical outcomes of AGB patients and investigate whether these measures can shorten hospital stays, reduce blood transfusions, and lower mortality rates, among other factors.

Our research findings indicate that professional nursing interventions are associated with favorable outcomes in AGB patients. Patients who received professional nursing interventions showed a significant increase in hemoglobin levels and a notable decrease in INR values, suggesting improved control over bleeding and coagulation abnormalities. These results are consistent with previous research; for instance, Jiang et al.^13^ studied the impact of nursing interventions on obese type 2 diabetes patients and found that patients receiving nursing interventions experienced improved hemoglobin levels and enhanced blood glucose management. However, our study emphasizes the importance of closely monitoring vital signs and administering timely medication to improve the prognosis of AGB patients.

Importantly, patients who received professional nursing interventions had shorter hospital stays, reduced blood transfusion requirements, and lower rates of rebleeding. The shorter hospitalization duration suggests that professional nursing interventions promote rapid recovery and effective management of AGB. Moreover, the decreased need for blood transfusions in the specialized nursing intervention group indicates effective hemostasis and bleeding management, potentially reducing complications and improving patient outcomes. The lower rebleeding rate in the specialized nursing intervention group further emphasizes the potential benefits of skilled nursing in preventing rebleeding. Early identification of bleeding and timely interventions may help minimize the risk of rebleeding in such patients. Prior research by Qing et al.^14^ on emergency nursing for patients with primary hepatocellular carcinoma rupture and bleeding showed a significant decrease in complication rates and mortality in patients who received emergency nursing, along with shorter intraoperative bleeding volume and hospital stays. Similarly, in a historical matched study conducted by Lee et al.^15^ the addition of nurse consultants to five clinical specialties had a significant impact on patient health and service outcomes in those departments, leading to reductions in emergency complications, shortened hospital stays, and improved patient satisfaction. The favorable results of the aforementioned nursing interventions align with our study's findings, and therefore, we recommend implementing specialized nursing interventions for emergency AGB patients.

Multivariate analysis confirmed that even after accounting for potential confounding factors such as age, gastrointestinal disease history, and severity of bleeding, specialized nursing interventions remained a significant predictor of rebleeding. This further strengthens the evidence of the positive impact of skilled nursing in managing AGB. ROC curve analysis demonstrated that specialized nursing interventions exhibited moderate to high discriminatory ability in predicting short-term clinical outcomes such as 30-day mortality, 90-day mortality, and readmission rates. Although the AUC values did not approach 1.0, indicating imperfect discriminative ability, they still highlighted the potential role of nursing interventions in assessing and improving the prognosis of AGB patients. Just as Morita et al.^16^ found that advanced practice nursing can significantly reduce the 30-day mortality rate in critically ill patients on mechanical ventilation, the authors recommend increased utilization of advanced practice nursing in adult ICUs.

Our study has several limitations that should be considered when interpreting the results. First, the study's retrospective nature may introduce bias and limit the ability to establish causality.

Furthermore, the sample size is relatively small, and the study was conducted at a single center. Additionally, there is a relatively high number of independent variables, posing a risk of overfitting, which may impact the generalizability of the study findings to other centers. Therefore, more prospective studies with larger sample sizes and multi-center collaborations are needed to strengthen the evidence and enhance the generalizability of the results. Furthermore, the specific details of the nursing interventions provided to patients were not explicitly captured, and the extent to which different interventions contributed to the observed outcomes remains unclear.

## Conclusion

In conclusion, our retrospective analysis underscores the positive impact of skilled nursing care on clinical outcomes for acute gastric bleeding (AGB) patients. Nursing interventions, vital in early bleeding identification and management, lead to shorter hospital stays, reduced blood transfusions, and lower rebleeding rates. This study sets the groundwork for evidence-based nursing practices to enhance AGB patient prognosis.

## Data Availability

The datasets used and analyzed during the current study are available from the corresponding author on reasonable request.
